# Adolescent Youth Survey on HIV Prevention and Sexual Health Education in Alabama: Protocol for a Web-Based Survey With Fraud Protection Study

**DOI:** 10.2196/63114

**Published:** 2025-01-29

**Authors:** Henna Budhwani, Ibrahim Yigit, Josh Bruce, Christyenne Lily Bond, Andrea Johnson

**Affiliations:** 1 Institute on Digital Health and Innovation, College of Nursing Florida State University Tallahassee, FL United States; 2 Birmingham AIDS Outreach Birmingham, AL United States

**Keywords:** HIV knowledge, PrEP, pre-exposure prophylaxis, adolescents, teenagers, transgender, MSM, men who have sex with men, south, bot protection, bots, fraud protection, survey protocol

## Abstract

**Background:**

In Alabama, the undiagnosed HIV rate is over 20%; youth and young adults, particularly those who identify as sexual and gender minority individuals, are at elevated risk for HIV acquisition and are the only demographic group in the United States with rising rates of new infections. Adolescence is a period marked by exploration, risk taking, and learning, making comprehensive sexual health education a high-priority prevention strategy for HIV and sexually transmitted infections. However, in Alabama, school-based sexual health and HIV prevention education is strictly regulated and does not address the unique needs of sexual and gender minority teenagers.

**Objective:**

To understand knowledge gaps related to sexual health, HIV prevention, and pre-exposure prophylaxis (PrEP), we conducted the Alabama Youth Survey with individuals aged 14-17 years. In the survey, we also evaluated young sexual and gender minority individuals’ preferences related to prevention modalities and trusted sources of health information.

**Methods:**

Between September 2023 and March 2024, we conducted a web-based survey with 14- to 17-year-olds who are assigned male at birth, are sexually attracted to male youth, and lived in Alabama. Half of the study’s participants were recruited through community partners, the Magic City Acceptance Academy and Magic City Acceptance Center. The other half were recruited on the web via social media. A 7-step fraud and bot detection protocol was implemented and applied to web-based recruitment to reduce the likelihood of collecting false information. Once data are ready, we will compute frequencies for each measure and construct summary scores of scales, such as HIV and PrEP knowledge, to determine internal consistency. Using multivariable logistic regression, we will examine associations between personal characteristics of survey respondents and key constructs using SPSS 29 (IBM Corp) or SAS 9.4 (SAS Institute).

**Results:**

Analyses are ongoing (N=206) and will conclude in June 2025. Preliminary results include a sample mean age of 16.21 (SD 0.88) years; about a quarter identified as transgender or gender nonconforming, with 6% stating their gender as a transgender woman. A total of 30% self-reported their race as African American or Black; 12% were Hispanic or Latinx. More than half reported being sexually active in the past 6 months. Primary data analyses will be completed in mid-2025. If findings are promising, results will be used as preliminary data to support the development of an intervention to address knowledge gaps and prevention preferences.

**Conclusions:**

If the study is successful, it will yield information on HIV knowledge, PrEP awareness, PrEP preferences, and related outcomes among sexual and gender minority teenagers in Alabama, an underserved, hard-to-reach, but also high-priority population for public health efforts to Ending the HIV Epidemic.

**International Registered Report Identifier (IRRID):**

DERR1-10.2196/63114

## Introduction

### Background

In Alabama, the undiagnosed HIV rate is over 20%; youth and young adults, particularly those who identify as sexual and gender minority individuals, are at elevated risk for HIV acquisition and are the only demographic group in the United States with rising rates of new infections [1,2]. Adolescence is a period marked by exploration, risk taking, and learning [3], making comprehensive sexual health education, typically delivered in school systems, a high-priority prevention strategy for HIV and sexually transmitted infections [4,5]. However, in Alabama, school-based sexual health education is strictly regulated [6]; school-based sexual health education must adopt an abstinence orientation, and HIV prevention is typically discussed within the context of heterosexual marriage [7,8]. In response to these structural limitations, community-based organizations have developed their own sexual health and HIV prevention education programs [9]. However, their content is typically delivered inconsistently, relies on funding availability, and is offered only to youth engaged in services. In 2019, pre-exposure prophylaxis (PrEP) was established as safe and effective in preventing HIV among adolescent-aged men who have sex with men (MSM), and the US Food and Drug Administration approved PrEP for individuals weighing at least 77 pounds (~34.9 kg), thereby making it available to adolescents [10,11]. Nevertheless, prescribing PrEP to adolescents remains under ideal levels [12]. Potentially, due to the lack of comprehensive HIV prevention and sexual health education in Alabama schools, there are high rates of HIV among sexual and gender minority youth and low rates of PrEP uptake. This leads to unanswered questions about what adolescents know or do not know about HIV and sexually transmitted infections (STIs) and about characteristics of their psychosocial profile, which may influence the acceptance of PrEP. Furthermore, new PrEP modalities are being developed, and clinicians and researchers alike are interested in learning about what PrEP options may be more acceptable to adolescents.

While the National Institutes of Health–funded Adolescent Medicine Trials Network for HIV/AIDS Interventions and HIV Prevention Trials Network have supported studies that assess PrEP preferences among sexual and gender minority adolescents and young adults [13,14], the age range usually begins at 15 or 16 years, with few studies enrolling as young as 14 years, even though sexual debut is thought to be younger in MSM as compared to the public [15]. There also continues to be a need to examine PrEP preferences of sexual and gender minority adolescents in the southern United States, where stigma related to HIV, sexual orientation, and gender identity is high and resources are limited [16,17]. Some research on adolescent knowledge and preferences related to HIV prevention has been conducted in the region [18,19]; however, studies more often occur with adolescents’ support persons, including guardians and providers, rather than the adolescents themselves and occur in clinical settings. Web-based surveys offer adolescents a comfortable and confidential way to share their essential opinions without feeling judged, especially since they heavily rely on digital technologies to build their social networks, receive social support, and obtain health information [5,20-22]. Engaging sexual and gender minority adolescents in research to understand their HIV and STI prevention is critical to reducing rates of HIV transmission but is challenging to do, making this protocol a high public health priority project.

While increasing PrEP uptake among young sexual and gender minorities is urgently warranted [23], and federal and local agencies are supporting behavioral interventions to reach this group [24], adolescent sexual and gender minority individuals will not engage in HIV biomedical prevention if (1) they do not know about PrEP, (2) PrEP options are not acceptable for their developmental period, or (3) they do not understand their risk [25-27]. There is an unmet need to deliver tailored HIV prevention for diverse sexual and gender minority youth, built upon adolescents’ existing knowledge and preferences [28,29]. To understand existing knowledge gaps and specifically address them via intervention, we conducted the Alabama Youth Survey to evaluate 14- to 17-year-old sexual and gender minority individuals’ preferences and knowledge related to HIV prevention, PrEP, and STIs. The aim of this study was to elucidate knowledge, beliefs, and preferences related to HIV and STI prevention from sexual and gender minority adolescents in Alabama through a web-based survey to inform future intervention development. Because this study is exploratory, no hypotheses are proposed.

### Objectives

In this study, we aim to elucidate knowledge, beliefs, and preferences related to HIV and STI knowledge and prevention among sexual and gender minority adolescents in Alabama through a web-based survey to inform future intervention development. If this study yields informative results, we will develop or adapt digital health intervention modules [30,31], a preferred and effective modality for reaching youth to address PrEP uptake while considering preferences and levels of knowledge.

## Methods

### Ethical Considerations

All study materials and procedures were reviewed and approved by the University of Alabama at Birmingham Institutional Review Board (IRB; IRB-300009255) and the Florida State University IRB (STUDY00003480). Informed assent was collected digitally from all study participants before data collection. Potential participants were informed of the purpose of the survey and anonymity, confidentiality, and voluntary principles before responding. We received parental waivers from both reviewing IRBs due to the precarious situation of many sexual and gender minority youth in Alabama. Still, an information sheet for parents was made available. Participation in the study was voluntary. Study data have been deidentified and stored on a secure server. Study participants received an incentive of a US $35 digital gift card.

### Eligibility Criteria

All potential participants completed a web-based screening survey via Qualtrics to verify their eligibility. To be eligible for the study, potential participants must have been 14-17 years old, assigned male at birth, report sexual attraction to men, and live in Alabama. MSM, transgender women, and genderqueer individuals were eligible if they met the aforementioned criteria.

### Recruitment

We partnered with three local agencies for this study. The Magic City Acceptance Academy is the only trauma-informed charter school for LGBTQIA+ (lesbian, gay, bisexual, transgender, intersex, queer/questioning, asexual) individuals in the US Deep South. Magic City Acceptance Academy recruitment was supplemented by recruitment from the Magic City Acceptance Center [32,33]. While both agencies are independent, they have ties to leadership and are connected to Birmingham AIDS Outreach [8,34]. About half of our sample was recruited in person from these sites. The other half was recruited on the web via social media through targeted advertisements on Facebook, Instagram, and Snapchat. Adolescents could access the survey via QR codes linked to an eligibility screener. Eligible individuals were automatically redirected to the Alabama Youth Survey.

### Bot Detection and Data Protection

Fraudulent data and fake responses are commonplace in web-based surveys, especially when a monetary incentive is provided. Examples of data fraud include bad actors, such as eligible individuals who submit surveys multiple times for multiple incentives, ineligible individuals who lie to meet eligibility criteria, and programmed bots. Since half of our sample was recruited on the web, we used extensive screening protocols, including (1) requiring the answering of youth-focused qualitative questions that require a typed response that would indicate residence, such as “What’s your favorite local restaurant?” and “What’s your favorite television show?” during screening [15]; (2) embedding multiple reCAPTCHAs throughout with review of Qualtrics bot detection; (3) requiring a US-based IP address; (4) locking surveys to disallow multiple submissions from the same IP address or device; (5) requiring all eligible respondents to provide an in-state phone number, which was then pinged for SMS text messaging verification before sharing the survey link—while many people have out-of-state area codes, this is much less likely for 14- to 17-year-old adolescents; (6) checking that all linked surveys were completed sequentially; and (7) using Zoom (Zoom Video Communications)–based verification audits when data seemed suspicious. Suspicious activities or indicators included but were not limited to submitting more than two surveys back to back, surveys received from the same small town in Alabama, and using an AOL or Yahoo (Yahoo Inc) email address. We also changed the survey links weekly to avoid improper circulation. These procedures were completed for all participants who were recruited on the web to ensure data integrity.

### Adolescent Youth Survey

The Adolescent Youth Survey was developed and implemented following the CHERRIES (Checklist for Reporting Results of Internet E-Surveys) checklist [35]. The web-based survey was programmed in Qualtrics to collect data on HIV knowledge, perceived HIV risk, actual HIV risk, PrEP knowledge, preferences related to PrEP modalities, stigmas, and characteristics that are known to be related to HIV prevention among sexual and gender minority individuals. Some constructs were explicitly developed for this study, such as PrEP modality preference, while in other cases, we used validated scales. The measures are shown in Table 1 below. The question order was not randomized. Questions were adaptive, and questions that were not applicable were not presented. For example, if a participant was not sexually active, questions about using protection during sexual intercourse were not presented. Respondents were allowed to go “back” in the survey. However, they could not adjust their responses after pressing the final submit. We aimed to recruit 200 participants and were able to recruit a total of 206 participants between September 2023 and March 2024 via a convenience sample that was shared by those recruited via the Magic City Acceptance Academy, the Magic City Acceptance Center, and the web.

**Table 1:** Measures collected

Construct	Description or examples	Questions, n
Demographics	Age, gender, sex, race, ethnicity, sexual orientation, residence in Alabama, parental education, and income	10
HIV risk and prevention	Sexually active [36,37], injectable substance use, on PrEP^a^, and previous STI^b^ diagnosis [38]	10
HIV knowledge	HIV-KQ-18^c^ [39]	18
STI knowledge	STI Knowledge Scale [40]	27
Medication experience and preferences	Oral pills, shots, suppositories, patches, etc	13
PrEP familiarity	Seen commercials, types of PrEP, and PrEP brands	6
PrEP awareness	PrEP-COL^d^ Scale [41]	10
PrEP preferences	PrEP modalities, PrEP delivery locations, etc	20
Trusted sources	Types of information sources and people	24
Stigma	IHP-R^e^ [42] and Everyday Discrimination Scale [43]	14
Depression	PHQ-8^f^ [44]	8
Resilience	MOS Social Support Survey [45], GSE^g^ [46]	29

^a^PrEP: pre-exposure prophylaxis.

^b^STI: sexually transmitted infection.

**^c^**HIV-KQ-18: HIV knowledge questionnaire.

^d^PrEP-COL: PrEP Columbia Scale

^e^IHP-R: Revised Internalized Homophobia Scale.

^f^PHQ-8: Personal Health Questionnaire Depression Scale.

^g^GSE: General Self-Efficacy Scale.

### Pilot Testing

Study team members aged 20-24 years pilot-tested the survey multiple times to assess its completion time. It was determined to be about 20-25 minutes, depending on how slowly one reads the questions and the time to consider response options. We then pilot-tested the survey with the first 10 respondents and found similar completion metrics, with no complaints or concerns reported.

### Data Storage and Analysis

Encrypted data are saved on a Florida State University server with password protection. The database and associated data structures were developed before survey distribution and were not adjusted during the protocol. We asked participants to voluntarily provide emails and cell phone numbers to facilitate the provision of incentives. These personal data were only known to the incentive processors and were kept separate from survey responses. During data collection, we examined data quality weekly (eg, missing data, assessment of distributional assumptions, and identification of outliers) and will do so before statistical analysis is conducted. As a pilot study, missing scale data will not be estimated. Once data are ready, we will compute frequencies for each measure and for each scale to assess variability and internal reliability. We will construct summary scores of scales, such as HIV and PrEP knowledge, to determine if these have adequate internal consistency (Cronbach α≥0.70) [47]. Using multivariable logistic regression, we will examine associations between the personal characteristics of survey respondents and key constructs. We will use SPSS 29 (IBM Corp) or SAS 9.4 (SAS Institute) for quantitative analyses.

## Results

While this study was approved in 2021, data collection began in September 2023 and concluded in March 2024. Since the survey was open, the system could not record the number of unique visitors; thus, a formal response rate cannot be calculated. Data analysis is underway and will conclude in June 2025. The sample included 206 participants aged 14-17 years with a mean age of 16.21 (SD 0.88) years; about a quarter identified as transgender or gender nonconforming, with 6% explicitly stating their gender as a transgender woman. A total of 30% self-reported their race as African American or Black; 12% were Hispanic or Latinx. About half of them reported being sexually active in the past 6 months.

## Discussion

### Principal Findings

In this study, we aim to elucidate knowledge, beliefs, and preferences related to HIV and STI prevention among sexual and gender minority adolescents in Alabama through a web-based survey to inform future intervention development. If this study yields informative results, data could inform the adaptation or creation of a sexual health and HIV prevention intervention for sexual and gender minority adolescents who live in southern states, where school-based sexual health education may be unavailable or strictly limited.

While hypotheses were not proposed, based on preliminary analyses and the extant literature, we anticipate finding low levels of knowledge and high levels of poor mental health outcomes. Little is known about PrEP modality preference in this population, so we cannot anticipate findings related to this topic. Findings will provide intervention targets (eg, PrEP education) and the needed dose; very low knowledge will necessitate more significant emphasis on PrEP education. When recruiting on the web, we encountered an enormous amount of fraud and ultimately needed to implement SMS text messaging verification, wherein eligible participants would only be routed to the survey after their unique phone number, with in-state area code, was verified. Even after verification, since Google phone numbers can be created, we audited suspicious submissions, such as surveys that were completed too quickly. A total of 10% of web-recruited submissions were audited via a Zoom call with a trained study coordinator. The web-based survey and fraud detection strategies were feasible; however, completing this survey with data protection and scientific integrity was much more costly and time-intensive than expected. For future web-based survey studies, we recommend implementing SMS text messaging verification at study onset with a random audit of 20% of records. Audits should include a video call (preferred) or a voice call (acceptable). Email verification, while simple, was not reliable, likely due to the ease of creating email accounts.

As noted in the *Introduction*, little is known about 14- to 17-year-olds’ knowledge, beliefs, and preferences related to HIV and STI prevention in the United States; thus, comparison to other studies is challenging. Recent research on these topics has been conducted in global settings with mixed findings [48-50]. A unique feature of our survey study is the assessment of PrEP modality preference, including options currently in clinical trials or may be available outside of the United States. Findings on modality preference have the potential to inform clinical care and how providers offer PrEP to sexual and gender minority adolescents.

While the study team was meticulous in bot detection and other fraud measures, some participants did not receive face-to-face interaction, and thus, those recruited on the web may be different from participants recruited from our local community partners. Second, the study may be susceptible to desirability bias, especially from participants recruited via in-person venues. While our sample is large for sexual and gender minority adolescents, a larger sample would increase generalizability. Some domains would have been valuable to assess, such as living circumstances and previous experiences with adverse life events; however, in pilot testing, adolescents felt the survey could not be lengthened.

### Conclusions

If the study is successful, we will yield information on HIV knowledge, PrEP awareness, PrEP preferences, and related outcomes among sexual and gender minority teenagers in Alabama, an underserved, hard-to-reach, but also high-priority population for public health efforts to Ending the HIV Epidemic. Data can inform the development of a culturally appropriate (for southern contexts, to be adolescent-friendly) and modular HIV prevention intervention, targeting behavior change related to HIV prevention for sexual and gender minority adolescents, that can be seamlessly integrated into amenable community settings in the southern United States.

## Abbreviations

IRB: institutional review board
LGBTQIA+: lesbian, gay, bisexual, transgender, intersex, queer/questioning, asexual
MSM: men who have sex with men
PrEP: pre-exposure prophylaxis
STI: sexually transmitted infection

## Data Availability

Data are available upon reasonable request through this study’s lead author (HB).
